# Transcranial direct current stimulation to facilitate neurofunctional rehabilitation in children with autism spectrum disorder: a protocol for a randomized, sham-controlled, double-blind clinical trial

**DOI:** 10.3389/fneur.2023.1196585

**Published:** 2023-06-15

**Authors:** Marcela O. Araujo, Priscila Tamplain, Natália A. C. Duarte, Andréa C. M. Comodo, Giselle O. A. Ferreira, Amanda Queiróga, Claudia S. Oliveira, Luanda A. Collange-Grecco

**Affiliations:** ^1^Human Movement and Rehabilitation, Post Graduate Program, Evangelic University of Goias, Anápolis, Brazil; ^2^Children's Rehabilitation Department, Follow Kids Child Neurorehabilitation Clinic, Rio de Janeiro, Brazil; ^3^Department of Kinesiology, University of Texas at Arlington, Arlington, TX, United States; ^4^Department of Child Neurofunctional Physiotherapy, Center of Pediatric Neurostimulation, São Paulo, Brazil

**Keywords:** autism spectrum disorder, gait, balance, child, physical therapy, transcranial direct current stimulation

## Abstract

**Background:**

Anodal transcranial direct current stimulation (tDCS) over the primary motor cortex and cerebellum is gaining prominence in the literature due to its potential to favor learning and motor performance. If administered during motor training, tDCS is capable of increasing the effect of training. Considering the motor impairment presented by children with Autism Spectrum Disorders (ASD), atDCS applied during motor training may contribute to the rehabilitation of these children. However, it is necessary to examine and compare the effects of atDCS over the motor cortex and the cerebellum on the motor skills of children with ASD. This information may benefit future clinical indications of tDCS for rehabilitation of children with ASD. The aim of the proposed study is to determine whether anodal tDCS over the primary motor cortex and cerebellum can enhance the effects of gait training and postural control on motor skills, mobility, functional balance, cortical excitability, cognitive aspects and behavioral aspects in children with ASD. Our hypothesis is the active tDCS combined with motor training will enhance the performance of the participants in comparison to sham tDCS.

**Methods and design:**

A randomized, sham-controlled, double-blind clinical trial will be conducted involving 30 children with ASD that will be recruited to receive ten sessions of sham or ten sessions of active anodal tDCS (1 mA, 20 min) over the primary motor cortex or cerebellun combined with motor training. The participants will be assessed before as well as one, four and eight weeks after the interventions. The primary outcome will be gross and fine motor skills. The secondary outcomes will be mobility, functional balance, motor cortical excitability, cognitive aspects and behavioral aspects.

**Discussion:**

Although abnormalities in gait and balance are not primary characteristics of ASD, such abnormalities compromise independence and global functioning during the execution of routine activities of childhood. If demonstrated that anodal tDCS administered over areas of the brain involved in motor control, such as the primary motor cortex and cerebellum, can enhance the effects of gait and balance training in only ten sessions in two consecutive weeks, the clinical applicability of this stimulation modality will be expanded as well as more scientifically founded.

**Clinical trial registration** February 16, 2023 (https://ensaiosclinicos.gov.br/rg/RBR-3bskhwf).

## 1. Introduction

Autism spectrum disorder (ASD) is a neurodevelopmental condition with varied manifestations in terms of behavioral characteristics and adaptive functioning (1, 2). Although behavioral abnormalities are the main characteristics of the disorder, the number of children with ASD who seek motor rehabilitation has been increasing due to the negative impact that problems in motor control have on the daily living of this population (3–5). Approximately 83% of children with ASD have difficulties with their motor skills, with changes observed in gross and fine motor activities (5, 6). Furthermore, studies indicated delays in the acquisition of motor marks and fundamental motor skills (7, 8) as well as deficits in postural stability, an altered gait pattern, motor coordination problems and altered movement velocity (9–12).

The involvement of dysfunctional patterns of brain and cerebellum activity is increasingly clear with regards to abnormalities in the motor control of children with ASD. Some studies have demonstrated imbalances between excitation and inhibition in synaptic transmissions (13–15), with the involvement of frontostriatal circuits and cerebellar regions, which play an important role in the processing of information from the vestibular system and the control of balance (16–20). Although the dysfunctional patterns of the structures affect body stability and stability of the head during movement and global mobility, these structures also participate in cognitive functions and communication. An example is the participation of cerebellar processing in proprioception and fine motor control as well as attention, decision making, language and affective regulation (21, 22).

Children with ASD have abnormalities related to learning in the acquisition, retention and transference of motor skills (5), which are directly related to neurological controls of movement. The reduction in the activation of neural circuits necessary for the execution of gross and fine motor activities can be considered as one of the factors that can compromise the result of rehabilitation of children with neurodevelopmental disorders (23). Neurofunctional training requires the active practice of deficient motor activities, so that the execution of the necessary movements to perform the trained activity has an impact on increased brain activation. However, promoting increased activation of dysfunctional brain areas remained a major challenge for physical rehabilitation for a long time. Currently, rehabilitation has an important therapeutic tool, which, when associated with neurofunctional motor training, is capable of optimizing training results (24).

Noninvasive brain stimulation, specifically transcranial direct current stimulation (tDCS), has attracted increasing attention in the scientific literature on therapeutic interventions for individuals with ASD (25–28). Transcranial direct current stimulation is known to induce lasting changes in cortical excitability and is a safe, accessible form of noninvasive brain stimulation that involves the administration of a low-intensity monophasic electrical current to the scalp using sponge-silicone surface electrodes moistened with saline solution (29, 30). Cortical modulation is dependent on the polarity of the current. Anodal stimulation increases cortical excitability, favoring the depolarization of the neuronal membrane, whereas cathodal stimulation exerts an inhibitory effect through hyperpolarization of the neuronal membrane (31, 32). tDCS provides a modulatory effect on cortical function, being easy to apply and at a lower cost when compared to other transcranial stimulation techniques (33). The benefits of tDCS include the possibility of use during the execution of different activities and motor tasks and combining it with other interventions (34, 35).

Considering its potential to favor learning and motor performance, the administration of anodal tDCS over the primary motor cortex and cerebellum has been gaining prominence in the literature (33, 34, 36). The development of gait and balance is linked to brain development, with the motor cortex and cerebellum considered fundamental structures (37, 38). The primary motor cortex is related to specific aspects of movement, such as direction, speed, acceleration and strength (39, 40). Anodal tDCS over this area of the brain has been performed in numerous studies involving individuals in all cycles of life, demonstrating that the promotion of the cortical modulation of neural networks has the potential to alter and favor learning and the consolidation of motor patterns, especially if stimulation occurs in synchrony with motor training (41, 42). Likewise, anodal tDCS over the cerebellum can result in faster, more effective adaptations in terms of locomotion and postural control. Anodal cerebellar stimulation is believed to facilitate the connectivity between the cerebellum and motor cortex through cerebellar-thalamocortical pathways (43, 44). Thus, the administration of electrical current could benefit motor control through direct and indirect effects in areas of the brain responsible for the control of body movements, short-term memory and postural control.

In the last decade, clinical trials have begun to demonstrate the promising effects of tDCS in the physical rehabilitation of children with motor impairments (45–50). Considering the pediatric population, children with cerebral palsy represent the most frequently studied population in studies on transcranial stimulation. These studies have shown that anodal tDCS over the motor cortex can manage effects on movement control, functional mobility, gait, gross motor function and balance (45, 46, 48–50). Likewise, the clinical trial analyzing the effects of anodal cerebellar tDCS demonstrated better effect on the spatio-temporal gait parameters and static balance of children with ataxic cerebral palsy (47). The effects were mainly observed when tDCS was applied during ten sessions (20 min) of motor training (51). The results on motor activities are directly related to the type of motor training, for example, if gait is trained during the application of anodic tDCS, the main results observed will be on gait and mobility (24). The target brain areas of transcranial stimulation that promoted positive effects on motor skills were the primary motor cortex, the area most frequently studied in children with motor disorders, and the cerebellum (51). Furthermore, predictive factors of positive tDCS responses were identified, the main ones being the presence of the motor evoked potential in the pre-intervention evaluation and subcortical encephalic lesions (24).

Recent evidence has demonstrated the promising results of tDCS for individuals with ASD, with a reduction in the intensity of the symptoms of the disorder, especially with regards to communication (nonverbal and social), a reduction in stereotyped movements and an improvement in working memory (28, 52, 53). However, clinical trials involving children with ASD have almost exclusively analyzed the effects of anodal tDCS over the left dorsolateral prefrontal cortex (54–57) or the temporoparietal junction with the aim of affecting dysfunctional areas directly related to the primary diagnostic characteristics of the disorder (compromised executive functions and social communication) (58). The effects of tDCS over the primary motor cortex during motor training were the focus of only one clinical trial identified to date, which reported promising results in terms of balance and motor skills in children with ASD (59). One study analyzed the effects of ten sessions of bilateral cerebellar anodal tDCS on the electroencephalographic activity of children with ASD. The results demonstrated that the active tDCS presented better results than those obtained with the placebo stimulation in the modulation and in the increase of the cerebral complexity. Increased complexity was observed in the cerebellar-cerebral circuitry, mainly in the left and right frontal cortical regions, the right central cortical region, and left parietal cortical region (60). However, this clinical trial exclusively analyzed the effects of tDCS on the motor cortex, and no studies were identified in the literature that analyzed the effects of tDCS on the cerebellum – the brain area that is related to the poor balance control shown by children with ASD.

As a resource considered safe, inexpensive and easy to administer, tDCS could contribute to the rehabilitation of these children, as its neurophysiological effects have been demonstrated to favor the learning of new voluntary motor and postural control strategies. Considering the complexity of brain activity dysfunctions observed in ASD, we need to investigate whether anodal tDCS may (or may not) contribute to motor training results. In addition, it is necessary to understand what are the effects of facilitating cortical excitability of fundamental structures of motor control, such as, for example, the primary motor cortex and the cerebellum. This information may benefit future clinical indications of tDCS for rehabilitation of children with ASD. However, the scientific literature is extremely limited regarding the effects of tDCS on areas of motor control in children with ASD.

The main goal of the present study is to compare the effects of ten 20-min sessions of neurofunctional motor training combined with anodal tDCS over the primary motor cortex, cerebellar region and sham stimulation on motor ability in children with ASD. Specifically, the aim is to determine whether anodal tDCS over these areas of the brain can optimize the effects of gait and postural control training on gross and fine motor skills, mobility, functional balance, motor cortical excitability, cognitive aspects and behavioral aspects in children with ASD. Our central hypothesis is that all individuals will present functional improvements following gait and balance training, but the benefits will be more evident after the administration of active tDCS (primary motor cortex and cerebellar region) compared to the group receiving sham tDCS due to the neurophysiological effects on cortical excitability. Moreover, we believe that the effects of anodal tDCS over the primary motor cortex will be restricted to motor outcomes, whereas the effects of anodal stimulation over the cerebellar region will also be found in outcomes related to cognitive and behavioral aspects.

## 2. Methods

We Registered this trial on The Brazilian Registry of Clinical Trials (RBR-3bskhwf). This paper has been reported in accordance with the standard Protocol Items: Recommendations for Interventional Trials (Spirit) (61). The study was reviewed and approved by Ethical Committee of the Evangelic University of Goias, under the number CAAE: 64474922.0.0000.5076. Written informed consent to participate in this study was provided by the participants’ legal guardian/next of kin.

### 2.1. General study design

**Figure 1:**
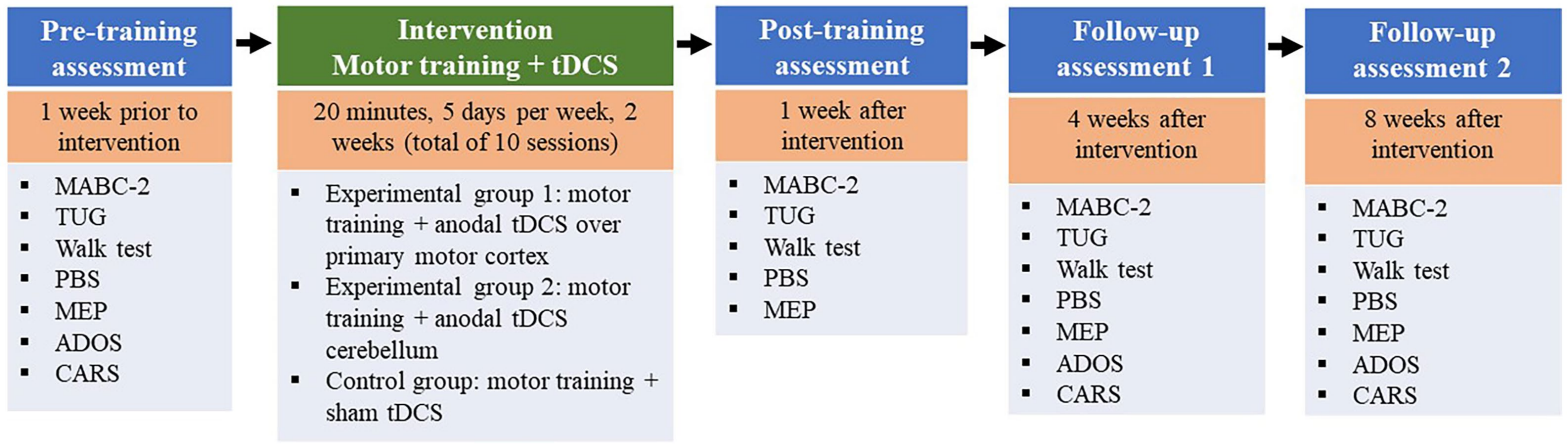
List of specific time points on this line. Motor training associated with tDCS (active or sham) at a frequency of five sessions per week for two consecutive weeks (total of ten sessions). MABC-2, Movement Assessment Battery for Children-2; PBS, Pediatric Balance Scale. TUG, Timed Up and Go; ADOS, Autism Diagnostic Observation Schedule; CARS, Childhood Autism Rating Scale.

A randomized, sham-controlled, double-blind study will be conducted involving children with a diagnosis of ASD. We will compare three montages: anodal tDCS over the primary motor cortex, anodal tDCS over the cerebellar region and sham stimulation. Stimulation will be administered in all three groups during 20 min of motor training focused on gait and balance training (Figure 1).

The study will involve the following visits:

- Visit 1: Screening of participants according to the eligibility criteria and signing of the statements of consent.
- Visits 2 and 3: Initial assessment (pre-intervention) one week prior to the onset of the intervention, with the investigation of motor ability, mobility, functional balance, motor cortical excitability, cognitive aspects and behavioral aspects. After the initial assessment, the participants will be randomly allocated to the different intervention groups. These visits will be held on two non-consecutive days and will last a maximum of 1.5 h.
- Visits 4 to 14: Ten intervention sessions in accordance with the allocation to the different groups. Sessions will be held for two consecutive weeks at a frequency of five sessions per week (Monday to Friday) with a maximum duration of approximately 45 min, 20 min of which will be tDCS combined with motor training.
- Visits 15 and 16: Post-intervention assessment one week after the end of the intervention with the investigation of motor ability, mobility, functional balance and motor cortical excitability. These visits will be held on two non-consecutive days and will last a maximum of 1.5 h.
- Visits 17 and 18: First follow-up assessment four weeks after the end of the intervention, with the investigation of motor ability, mobility, functional balance, motor cortical excitability, cognitive aspects and behavioral aspects. These visits will be held on two non-consecutive days and will last a maximum of 1.5 h.
- Visits 19 and 20: Second follow-up assessment eight weeks after the end of the intervention, with the investigation of motor ability, mobility, functional balance, motor cortical excitability, cognitive aspects and behavioral aspects. These visits will be held on two non-consecutive days and will last a maximum of 1.5 h

**Figure 2:**
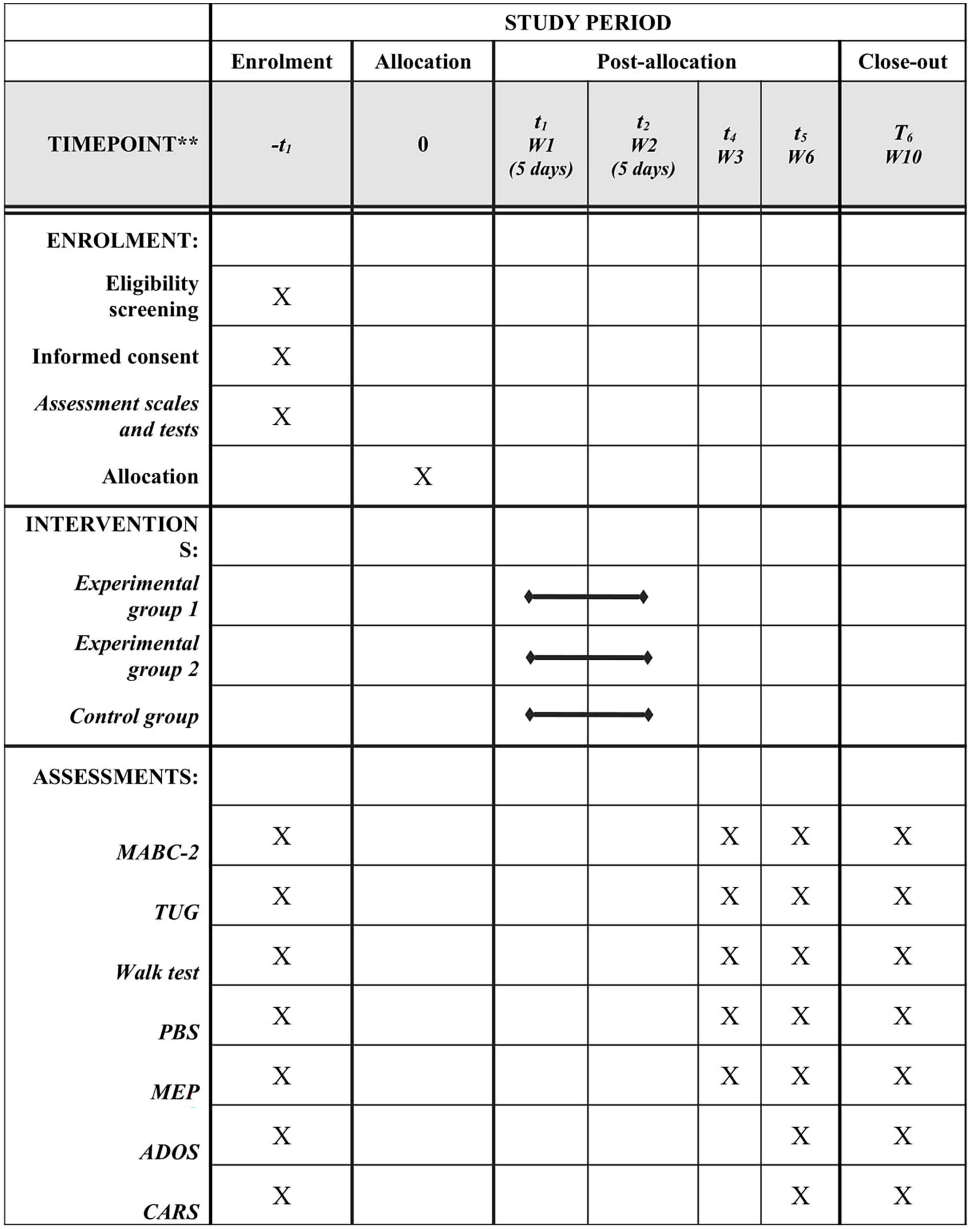
SPIRIT: Description of protocol of study, timeframe of enrolment, interventions and assessments. MABC-2, Movement Assessment Battery for Children-2; TUG, Timed Up and Go. PBS: Pediatric Balance Scale; MEP, Motor Evoked Potential; ADOS, Autism Diagnostic Observation Schedule; CARS, Childhood Autism Rating Scale.

Figure 2 displays the flowchart of the study in accordance with the SPIRIT statement (61).

### 2.2. Participants

Thirty children will be recruited from the XXX Child Neurorehabilitation Clinic in the city of XXX, XXX. Participants will undergo detailed screening with the eligibility criteria for inclusion in the study.

The sample size was calculated considering the primary clinical results, which are the total and balance score of the Movement Assessment Battery for Children, 2nd ed. (MABC-2) (62). The calculation of the sample size was based on data from a study involving a group of individuals with ASD who received motor training combined with tDCS over the primary motor cortex (59). The estimated effect size of the previous study was 1.39 (change in total MABC-2 score of 16.5 with a standard deviation [SD] of 5.98; change in score of balance category of MABC-2 of 2.4, SD: 1.70; α = 0.05 (two-tailed) and β = 0.8). The estimate indicated a minimum of 24 participants (eight per group). Considering a possible dropout rate of 20%, 30 participants will be recruited.

The children will be screened for participation in the study based on the following:

Inclusion criteria: 1) Diagnosis of ASD confirmed though a clinical examination; 2) Standard neurobehavioral assessment that confirms and categorizes the severity of ASD; 3) Levels I, II and III according to the Diagnostic and Statistical Manual of intellectual disability (DSM-5); 4) Age three to eight years; 5) Sufficient response to understand simple verbal commands; 6) No change in medication in the six months prior to the onset of the study or during the study; 7) Signed statement of informed consent by a legal guardian; 8) Agreement to participate in study through term of assent.

Exclusion criteria: 1) Diagnosis of epilepsy or seizure within the 12 months prior to the onset of the study; 2) Neurological, neuromuscular disorders or syndromes other than ASD; 3) Having been submitted to orthopedic or neurological surgery within 12 months prior to the intervention; 4) Orthopedic deformities with indication for surgery; 5) Implants in the skull or having undergone some neurosurgical procedure; 6) Metallic implants in the head, neck, chest or upper limb; 7) Hearing devices; 8) Degree of cooperation incompatible with the adequate performance of the activities proposed in the present project.

Withdrawal criteria: Participants who decline to continue in the study, those not present on the days of the experiment and those who miss up to two of the 10 intervention sessions will be removed from the study.

**Randomization procedure:** Randomization will be performed using a program for the generation of random numbers to allocate the participants to one of the following interventions:

- Experimental group 1 – Motor cortex stimulation: anodal tDCS over the primary motor cortex combined with neurofunctional motor training;
- Experimental group 2 – Cerebellar stimulation: anodal tDCS over the cerebellar region combined with neurofunctional motor training;
- Control group – Sham stimulation: sham tDCS combined with neurofunctional motor training.

Randomization will be performed considering stratification based on the severity of ASD according to the Brazilian version of the Childhood Autism Rating Scale (CARS-BR) (63). The randomization sequence will be generated for each category (mild, moderate and severe) with adequately organized distribution in sequentially numbered opaque envelopes.

**Blinding:** After the inclusion of participants based on the eligibility criteria and after the pre-intervention assessment, the allocation of the participants will be informed to the researcher who will carry out the intervention. The researcher in charge of the randomization process will not participate in any other aspect of the study and will not have access to the participants. The researchers involved in the assessment procedures, the participants and their families will not be aware of the allocation of the participants. This information will be provided to the families and participants after the complete conclusion of the procedures of the study. The investigator in charge of the interventions will not be blinded to the allocation of the participants, as it will be necessary to select the active or sham stimulation option of the equipment.

### 2.3. Assessment measures

The study will involve assessment tools used for the diagnosis and classification of the severity of ASD, with the measurement of cognitive and behavioral aspects, an assessment tool for gross and fine motor skills (primary outcome), a functional mobility test, a test for the assessment of spatiotemporal gait variables, a functional balance scale, a neurophysiological assessment of motor cortical excitability (evoked motor potential) and a questionnaire addressing side effects. In addition, informations about the medications will be collected, including the name of the medication, dosage and frequency of use, and how long the child has been using the medication. The order of evaluations will be random, through simple randomization carried out by draw.

#### 2.3.1. Assessment tools and categorization of groups according to ASD

The Autism Diagnostic Observation Schedule (ADOS) will be used to confirm the diagnosis of ASD and to assess the behavior of the children in terms of particular structured activities. The ADOS is composed of four modules, all with good reliability, sensitivity and specificity for ASD (64, 65). Each module is designed for individuals with specific degrees of linguistic development and involves a protocol of structured, dynamic interactive activities conducted by a trained examiner in a period of approximately 45 min. The aim of the different tasks is to provide information on social behavior, communication and general aspects of the individual being assessed. Based on observations on how the individual performs the tasks, the therapist answers a questionnaire, attributing scores for the different behaviors. The questions are organized on a four-point scale, with 0 indicating no abnormality and 3 indicating moderate to severe abnormalities (66). To be classified as an individual with autism or ASD according to the ADOS, the sum of the scores defined in the algorithms of the four modules must reach minimum cutoff points in the domains referring to communication and reciprocal social interaction (67, 68). The ADOS is an ASD diagnostic instrument, but we also chose to use it in post-intervention assessments, with the intention of identifying possible changes in the participants’ performance during the execution of tasks.

The Brazilian version of the Childhood Autism Rating Scale (CARS-BR) will be administered to the parents or guardians for the classification of the severity of ASD. CARS-BR is an observation scale with 15 items to assist in the identification of children with ASD and distinguish children with impaired development without autism as well as distinguish autistic children with intellectual disability. Its importance consists of the differentiation of mild to moderate autism from severe autism. The scale is short and appropriate for use on any child older than two years of age. The scale was validated for Brazilian Portuguese in a study conducted for 15 years involving 1,500 children with autism (62). The scale assessed behavior in 14 domains generally affected by autism plus a general autism impression category. The scores of each domain range from 1 (within the limits of normality) to 4 (severe autistic symptoms). The overall score ranges from 0 (no characteristics of autism) to 60 (all severe characteristics met). Its use offers several advantages over other instruments, such as the inclusion of items that represent varied diagnostic criteria and reflect the actual dimension of the disorder, applicability to children of all ages (including preschoolers) and objective, quantifiable scores based on direct observation (62).

#### 2.3.2. Neurofunctional assessment measures

##### 2.3.2.1. Primary outcome

Gross and fine motor skills: Motor skills will be assessed using the Movement Assessment Battery for Children-2 (MABC-2). The principal aim for the use of this measure will be the assessment of balance using a valid measure for children with ASD (69, 70) employed in a previous clinical trial analyzing the effects of tDCS in this population (59). The MABC-2 comprises the categories of manual dexterity, aiming and catching and balance, with norms of the tests for three different age groups (3–6 years, 7–11 years and 12–17 years). The balance subscale has three items for each age group (one static and two dynamic tests). The standard score based on age is equivalent in percentile for each subscale to be transcribed from the raw score of the child. The percentiles are normally used to guide the interpretation when explaining the scores obtained on the test to parents and teachers, with the following zones: (a) Red zone – scores equal to or lower than the 5th percentile; (b) Amber zone – scores between the 5th and 15th percentile; and (c) Green zone – scores above the 15th percentile (69–71).

The MABC is used to identify motor difficulties in children and is particularly associated with the identification of Developmental Coordination Disorder (DCD). The assessment has been used and validated with several atypical populations, including those with ASD. Since there is no recommendation or guideline for evaluation of motor ability in individuals with ASD, the MABC was an optimal choice for the present study. Several studies have shown that individuals with ASD have motor difficulties consistent with DCD. In Miller et al. (72), over 90% of the cases in the ASD group met criteria for co-occurring DCD. In a recent systematic review, the majority of articles (92.5%) indicated that 50–88% of children with ASD had significant motor impairments on standardized motor assessments and/or functional questionnaires. The nature of motor and function problems in ASD were consistent with DCD (6).

##### 2.3.2.2. Secondary outcomes

Functional balance: The Pediatric Balance Scale will be used for the assessment of functional balance. This scale is composed of 14 items that address the performance of functional balance common to daily living. Each item is scored on a five-point scale ranging from 0 to 4, with a maximum total score of 56 points. The points are based on the time in which a position is maintained, the distance to which an upper limb is capable of reaching in front of the body and the time required to complete the task. The children will perform the tasks barefoot. The average duration of test is 30 min (73, 74).

Functional mobility: The Timed Up and Go (TUG) test (75) will be performed with the aid of the inertial G-sensor (BTS Bioengineering), which is widely used for the assessment of functional mobility. The TUG test consists of the time in seconds required for the individual to stand up from a standard chair without using the arms, walking three meters, turning around, returning to the chair and sitting down again. The children will be instructed to perform the test safely at a self-selected pace. The test will be performed three times – the first two for familiarization and the third will be used for analysis. The variables obtained during the execution of the TUG test with the use of the G-sensor and analyzed in the study will be the duration of the phases and peak of the trunk in angular space: duration of the task (s), duration of the phase from sitting to standing (s), going gait (s), duration of turning phase (s), return gait (s), duration of final turning phase (s), duration of phase from standing to sitting (s), duration sitting to standing – peak trunk flexion (°), duration of sitting to standing – peak trunk extension (°), duration standing to sitting – peak trunk flexion (°), duration of standing to sitting – peak trunk extension (°). The average duration of the TUG test is 120 s.

Spatiotemporal gait variables: Spatiotemporal gait variables will be determined during the execution of the Walk Test (76). The participants will be instructed to remain standing and, when instructed, walk normally without running for ten meters, turn around a cone, return, turn around and return to the initial position. During the execution of the test, spatiotemporal gait variables will be collected by the inertial sensor (G-Sensor®, BTS Bioengineering S.p.A. Italy), enabling gait analysis with the Walk Test. The portable G-sensor is a wireless system of inertial sensors for human movement analysis. The sensors are controlled by a data recording unit (up to 16 elements) using ZigBee radio communication. Each sensor is 62 mm × 36 mm x 16 mm, weighs 60 g and is composed of a three-axis accelerometer (maximum scale: ± 6 g), a three-axis gyroscope (complete scale: ± 300°/s) and a three-axis magnetometer (complete scale: ± 6 Gauss). Only one sensor will be used in the study, with data collected at a sampling frequency of 50 Hz. The data from the inertial sensor will be transmitted *via* Bluetooth to a computer and processed using the BTS G-STUDIO, version 2.6.12.0, which automatically furnishes the variables. The participants will perform the test three times – two for familiarization and the third for analysis. The following variables will be collected: duration of the gait cycle (s), cadence (steps/min), velocity (m/s), stride length (m) of left and right lower limbs and step length (%) of left and right lower limbs. The procedure will last an average of 10 min.

#### 2.3.3. Neurophysiological assessment measure

Cortical excitability: Transcranial magnetic stimulation (TMS) will be used for the assessment of cortical excitability using a magnetic stimulator with a circular MagVenture coil. The response to stimulation applied to the motor cortex will be recorded in the thumb adductor and femoral quadriceps of both limbs to identify the motor evoked potential (MEP) and the motor cortical representation pattern (contralateral, bilateral or unilateral). The MEP responses will be filtered and amplified using a surface electromyograph. The signals will be transferred to a personal computer for offline analysis using data collection software. The motor threshold and MEP will be measured using the single-pulse TMS method. The motor threshold will be found in the region of the cortex with the lowest intensity necessary to generate a peripheral response. The same method will be employed to determine the MEP using 110% of the motor threshold intensity. Ten measures of the MEP will be performed in each assessment step.

#### 2.3.4. Adverse effects assessment measure

Potential adverse effects of tDCS will be investigated after each intervention using a questionnaire administered to the child. The children will be asked about the perception of any of the following symptoms during the session: tingling sensation, burning sensation, headache, pain at the sites where the electrodes were positioned, sleepiness and change of mood. The children will be instructed to answer using a three-point scale: 1 – “I did not feel it at any time during the session”; 2 – “I felt it at some times during the session”; 3 – “I felt it during the entire session.” When a child reports an adverse effect (answers 2 or 3), she or he will be asked to quantify the intensity (0 – none, 1 – weak, 2 – moderate, 3 – strong or 4 – unbearable). The children will be asked openly prior to each session about the occurrence of headache, pain on the scalp, burning sensation, tingling, redness of the skin, sleepiness, concentration difficulty or mood swings during periods between sessions. Considering the potential for impaired communication, the guardians will also be asked about their perceptions and the therapists in charge of the intervention will be instructed to observe and report behaviors suggestive of discomfort. The questionnaire has a maximum duration of 5 min for its application.

### 2.4. Intervention procedures

The ten intervention sessions will be held over 2 consecutive weeks, with five sessions per week (Monday to Friday). Upon arrival to the session, the participants and guardians will be oriented and prepared to undergo the intervention. The electrodes will be positioned and the child will perform 20 min of neurofunctional training combined with tDCS. At the end of each session, the child will answer the questionnaire on adverse effects. Neurofunctional training associated with tDCS will be performed by two investigators. One investigator will be responsible for conducting the neurofunctional training and the other for the tDCS, ensuring that the electrodes remain properly positioned during the neurofunctional training. In addition, the tDCS equipment used indicates the quality of contact between the electrodes and the cranial surface and the maintenance of current flow, indicating the intensity applied throughout the intervention period. Therefore, even minimal changes can be easily identified.

Transcranial direct current stimulation: Transcranial stimulation will be administered using the tDCS device (Soterix Medical Inc., USA) with two sponge (non-metallic) surface electrodes (5 × 7 cm2) moistened with saline solution. The children will be randomly allocated to three types of treatment: 1) anodal tDCS over the primary motor cortex; 2) anodal tDCS over the cerebellum; and 3) sham tDCS. Following the 10–20 International Electroencephalogram System (77), the group to receive anodal tDCS over the primary motor cortex will have the anode positioned over Cz and the cathode positioned over the right deltoid muscle, whereas the group to receive anodal tDCS over the cerebellum will have the anode positioned one centimeter below the inion and the cathode positioned over the central supraorbital region (47). For sham stimulation, all electrode placement procedures will be performed using the montage over the primary motor cortex.

The participants in the active tDCS groups will receive an electrical current at an intensity of 1 mA during the 20 min of neurofunctional training. The intensity of the current will be gradually increased in the initial 30 s, remain at 1 mA for 20 min and gradually reduced in the final 30 s. For the sham intervention, the current will be gradually increased to 1 mA in the initial 30 s, giving the children the initial sensation of the electrical current, but no stimulation will be administered for the rest of the session. This is considered a valid control procedure in studies involving tDCS (30).

Neurofunctional training: The training was carried out in a therapy room organized specifically for the intervention. Composed of postural control training and gait training. Immediately after the onset of tDCS (active or sham), the three groups will perform neurofunctional training consisting of postural control exercises and treadmill training conducted by a trained, experienced physiotherapist, with the participants performing the intervention individually. The 20 min of intervention will be divided into two steps (10 min each).

- Treadmill training: The training was performed on a Inbramed treadmill (Milenium ATL model, RS, Brazil). The treadmill training speed will be established based on the performance of the child in each session. Ideal speed will be the maximum speed at which the child is able to walk with adequate support of the feet upon initial contact and throughout the entire support phase of the gait cycle. The speed will be gradually increased during the initial two minutes and gradually reduced during the final two minutes, maintaining a constant speed in the interim. During training, the children used their habitual shoes, which were duly placed by the physiotherapist. The therapist remained behind the participants, facilitating gait training if necessary and ensuring both the safety of the intervention and the best possible movement kinematics. Throughout treadmill training, the therapist in charge also provided verbal commands to the participant to improve the execution of gait (48, 78).
- Postural control exercises: The second step will involve a circuit of activities based on the tasks of the MABC-2 to favor the improvement in postural control. The activities will be four exercises performed for 40 s with a 20-s rest interval. The four exercises will be selected individually according to the performance of each child during the MABC-2. Items from the balance and aim subscale will be defined as circuit exercises, which the child is able to perform with a delay (does not reach the complete performance of the item) and with qualitative observations in aspects of posture/body control. Some examples of activities are: balancing on one foot, walking on tiptoes, jumping, etc. During the execution of the circuit activities, the physiotherapist will remain beside the child, guaranteeing his/her safety in the face of imbalance and offering verbal commands with motivational phrases and guidance for the steps of the activities. Each participant will perform two complete circuits, with a two-minute rest interval between circuits.

### 2.5. Statistical analysis

The precision of all data will be verified by two researchers and a blinded statistician will conduct all analyses. The Kolmogorov–Smirnov test will be used to determine the distribution of the data regarding adherence to the Gaussian curve. Parametric data will be expressed as mean and standard deviation or 95% confidence interval. Nonparametric data will be expressed as median and interquartile range. Two-way analysis of variance (ANOVA) followed Tukey’s *post hoc* test will be used to assess the effects of tDCS on the outcome variables. For all analyses, the fixed independent variables will be group (active tDCS over the primary motor cortex, active tDCS over the cerebellum and sham tDCS) and assessment time (pre-intervention, post-intervention, follow-up 1 and follow-up 2). The dependent variables will be the outcomes studied. The effect size (Cohen’s d) will be calculated from the difference in the values obtained during the pre-intervention, post-intervention, first follow-up and second follow-up assessments comparing the three groups. Predictive response factors will be identified through logistic regression analysis. Logistic regression models will be performed with the outcomes: type of daily medication, ASD severity, MEP (presence or absence) and pre-intervention MABC-2 result. The level of significance will be set at 5% (*p* < 0.05) and Statistical Package for the Social Sciences (SPSS, version 21.0) will be used for the analyses.

### 2.6. Procedure for handling missing data

Statistical analysis will involve intention-to-treat analysis. In the occurrence of missing data during the study, the statistical analysis will be performed by carrying forward data from the previous observation to adjust for the missing data in the assessments after the intervention.

### 2.7. Data management

All data will be stored for three years after the conclusion of the study in an online databank. All electronic files related to the study will only be accessible to key personnel and all computers will be protected by passwords. Each participant will receive an exclusive identifier upon inclusion to the study that will be used for all documentation related to the study. The forms and files in print will be kept in a cabinet by the coordinator of the study locked in a secure office.

### 2.8. Trial status

Recruitment of the participants began in January 2023 and should end in December 2023. The conclusion of the study is predicted for March 2024.

### 2.9. Expected results

We believe that the three intervention groups will exhibit improvements in motor skills, mobility and functional balance directly related to gait and balance training. However, the effect size is expected to be larger in the experimental groups involving motor training combined with active tDCS (motor cortex and cerebellum) compared to the control group (sham tDCS) with regards to the motor outcomes. We also expect the effects obtained in the experimental groups to be maintained at the follow-up evaluations four and eight weeks after the end of the interventions. Lastly, we expect the motor effects to result in an improvement in the global performance of the children with ASD who participate in the study, including cognitive and behavioral aspects assessed using ADOS and CARS.

## 3. Discussion

Motor training combined with tDCS has been shown to be safe, viable and effective for children and adolescents with motor control disorders. Considering the results available in the scientific literature demonstrating the promising effects of anodal tDCS in children with ASD, especially aspects related to communication and behavior, it is of interest to expand the knowledge regarding the effects of tDCS also on motor skills in this population. If demonstrated that anodal tDCS administered over areas of the brain involved in motor control, such as the primary motor cortex and cerebellum, can enhance the effects of gait and balance training in only ten sessions in two consecutive weeks, the clinical applicability of this stimulation modality will be expanded as well as more scientifically founded.

Although abnormalities in gait and balance are not primary characteristics of ASD, such abnormalities compromise independence and global functioning during the execution of routine activities of childhood. Motor rehabilitation poses a challenge for therapists, families and patients, as children with ASD are often enrolled in behavioral treatment programs that require a considerable number of intervention hours per day. Thus, therapeutic possibilities with execution in focused, short-term programs that prove effective could be a favorable option for the treatment of abnormalities in motor skills.

Therefore, the present protocol for a randomized controlled clinical trial involves assessment measures of results with representativeness in the daily activities of children. Motor skills will be assessed using the MABC-2, which consists of items that measure gross and fine motor skills, to determine whether motor training (gait and balance) combined with anodal tDCS achieves positive results not restricted to the motor task being trained. Scales and tests will be used to measure the effects on mobility and balance during functional activities, such as walking, standing up, sitting down, changing direction, etc. These assessment tools will enable the understanding of the effects on an important domain of the International Classification of Functioning, Disability and Health – ACTIVITY, which is the primary focus of the proposed intervention.

The results of the clinical trial will also enable a better understanding of the effects of tDCS considering the specificity of the areas of the brain stimulated (primary motor cortex and cerebellum). Studies as that proposed in this protocol can also contribute to better decision making in terms of the “ideal target area” for the administration of tDCS in accordance with the clinical objective and proposed training.

Moreover, the proposed study involves the determination of the motor evoked potential, which is a measure of cortical excitability for the assessment of brain structure and function, enabling the analysis of whether a neurophysiological change in this parameter will be related to the results obtained in aspects related to motor skills – gait and balance. Lastly, the assessment of cognitive and behavioral aspects using the ADOS and CARS measures will enable understanding whether the intervention has an impact on the symptoms and intensity of the symptoms of ASD as well as indirectly enable measuring the effects of the intervention on the child’s participation, as these assessment measures involve the perceptions of family members with regards to the child’s performance and participation in routine activities.

## Ethics statement

The study was analyzed and approved by the Human Research Ethics Committee of Universidade Evangélica de Góias with the co-participation of the Follow Kids Child Neurorehabilitation Clinic (certificate number: 64474922.0.0000.5076). Written informed consent to participate in this study was provided by the participants’ legal guardian/next of kin.

## Author contributions

AC, GF, and AQ will coordinate communication with participants and collect data. MA, ND, CO, and LC-G contributed to all stages of this protocol including discussion on ideas, study design, and protocol. MA, PT, and LC-G drafted the manuscript and designed study outcome measures. All authors contributed to the article and approved the submitted version.

## Funding

The authors gratefully acknowledge financial support from the Brazilian fostering agencies Conselho Nacional de Desenvolvimento Científico e Tecnológico (CNPq, Nível 2. 2008–2020), Fundação de Amparo á Pesquisa do Estado de Goiás (FAPEG – PD&I 07/2020/ 202110267000212). The funders had no role in study design, data collection and analysis, decision to publish or preparation of the manuscript.

## Conflict of interest

The authors declare that the research was conducted in the absence of any commercial or financial relationships that could be construed as a potential conflict of interest.

## Publisher’s note

All claims expressed in this article are solely those of the authors and do not necessarily represent those of their affiliated organizations, or those of the publisher, the editors and the reviewers. Any product that may be evaluated in this article, or claim that may be made by its manufacturer, is not guaranteed or endorsed by the publisher.

## Abbreviations

ADOS: Autism Diagnostic Observation Schedule
ASD: Autism spectrum disorder
CARS-BR: Brazilian version of the Childhood Autism Rating Scale
DSM: Diagnostic and Statistical Manual of Mental Disorders
MABC: Movement Assessment Battery for Children
MEP: motor evoked potential
tDCS: transcranial direct current stimulation
TMS: Transcranial magnetic stimulation
TUG: Timed Up and Go
